# Phospholipid scramblase 1: a frontline defense against viral infections

**DOI:** 10.3389/fcimb.2025.1573373

**Published:** 2025-04-03

**Authors:** Alina X. Yang, Carmelissa Norbrun, Parand Sorkhdini, Yang Zhou

**Affiliations:** Department of Molecular Microbiology and Immunology, Brown University, Providence, RI, United States

**Keywords:** PLSCR1, antiviral, SARS-CoV-2, influenza A virus, HIV, Epstein-Barr virus, HCMV (human cytomegalovirus), HBV - hepatitis B virus

## Abstract

Phospholipid scramblase 1 (PLSCR1) is the most studied member of the phospholipid scramblase protein family. Its main function is to catalyze calcium (Ca^2+^)-dependent, ATP-independent, bidirectional and non-specific translocation of phospholipids between inner and outer leaflets of plasma membrane. Additionally, PLSCR1 is identified as an interferon-stimulated gene (ISG) with antiviral activities, and its expression can be highly induced by all types of interferons in various viral infections. Indeed, numerous studies have reported the direct antiviral activities of PLSCR1 through interrupting the replication processes of a variety of viruses, including entry of severe acute respiratory syndrome coronavirus 2 (SARS-CoV-2), nuclear localization of influenza A virus (IAV), and transactivation of human immunodeficiency virus (HIV), Epstein-Barr virus (EBV), human T-cell leukemia virus type-1 (HTLV1), human cytomegalovirus (HCMV) and hepatitis B virus (HBV). In addition to these direct antiviral activities, PLSCR1 also regulates endogenous immune components to defend against viruses in both nonimmune and immune cells. Such activities include potentiation of ISG transcription, activation of JAK/STAT pathway, upregulation of type 3 interferon receptor (IFN-λR1) and recruitment of Toll-like receptor 9 (TLR9). This review aims to summarize the current understanding of PLSCR1’s multiple roles as a frontline defense against viral infections.

## 1 Background

### 1.1 Maintenance of plasma membrane asymmetry

Phospholipids are the most fundamental components of lipid bilayers (Huang et al., 1964). They maintain the integrity of the biological membrane system, including the plasma membrane, nuclear membrane and membranes of intracellular organelles (Stillwell, 2016). There are 4 major phospholipids in the plasma membrane of animal cells: phosphatidylserine (PS), phosphatidylethanolamine (PE), phosphatidylcholine (PC) and sphingomyelin (SM) (Cooper, 2000). PS and PE are enriched in the inner leaflet, while PC and SM are predominant in the outer leaflet, creating a natural asymmetry of the plasma membrane (Cooper, 2000). This asymmetry is maintained through transbilayer lipid motion, facilitated by several transmembrane enzymes, namely flippases, floppases and scramblases. Flippases flip aminophopholipids (PS or PE) from the outer leaflet to the inner leaflet, while floppases flop cholinephospholipids (PC or SM) from the inner leaflet to the outer leaflet, both in an ATP-dependent manner. In contrast, scramblases catalyze calcium (Ca^2+^)-dependent, ATP-independent, bidirectional and non-specific translocation of phospholipids (Daleke, 2003).

Since Ca^2+^ serves as a second messenger in many cell types, scramblases are able to respond to various cell stimuli, such as coagulation, apoptosis and pathogenic infections (Zwaal et al., 2005), making them valuable targets for further research. For example, Ca^2+^ is crucial for platelet activation. An increase in cytosolic Ca^2+^ levels is necessary for platelet activation during hemostasis and thrombosis by driving conformational changes and degranulation (Varga-Szabo et al., 2009). In healthy platelets, Ca^2+^-induced PS exposure by scramblases creates a catalytic membrane surface that supports the assembly and activity of coagulation factor complexes (the tenase complex and the prothrombinase complex) (Rosing et al., 1985a). However, in a rare congenital bleeding disorder known as Scott Syndrome, a genetic mutation is believed to directly affect lipid scramblase or disrupt its Ca^2+^-induced activation pathway in platelets (Dachary-Prigent et al., 1997). Platelets from individuals with Scott Syndrome exhibit a significant deficiency in Ca^2+^-induced scramblase activity, leading to impaired PS transport to the outer leaflet of the cell membrane, which in turn reduces the activity of tenase and prothrombinase complexes (Rosing et al., 1985b). In addition to its role in coagulation, Ca^2+^-activated scramblases are essential for apoptosis. In cerebral ischemia-reperfusion injury, scramblases in neurons are activated by Ca^2+^ influx from the extracellular milieu, induced by transient receptor potential canonical 5 (TRPC5) Ca^2+^ channel (Guo et al., 2020). This activation leads to increased scramblase activity, and subsequent PS exposure on the outer leaflet of plasma membrane, providing a phagocytosis signal for microglia to engulf the apoptotic neurons. Finally, many pathogens, such as malaria-causing *Plasmodium falciparum*, can enhance Ca^2+^ influx into infected cells through non-selective Ca^2+^ channels, potentially activating scramblases (Lang et al., 2004). Scramblase-facilitated PS exposure may thus contribute to the clinical manifestations of various pathogenic infections.

### 1.2 Structure and functions of PLSCR1

**Figure 1:**
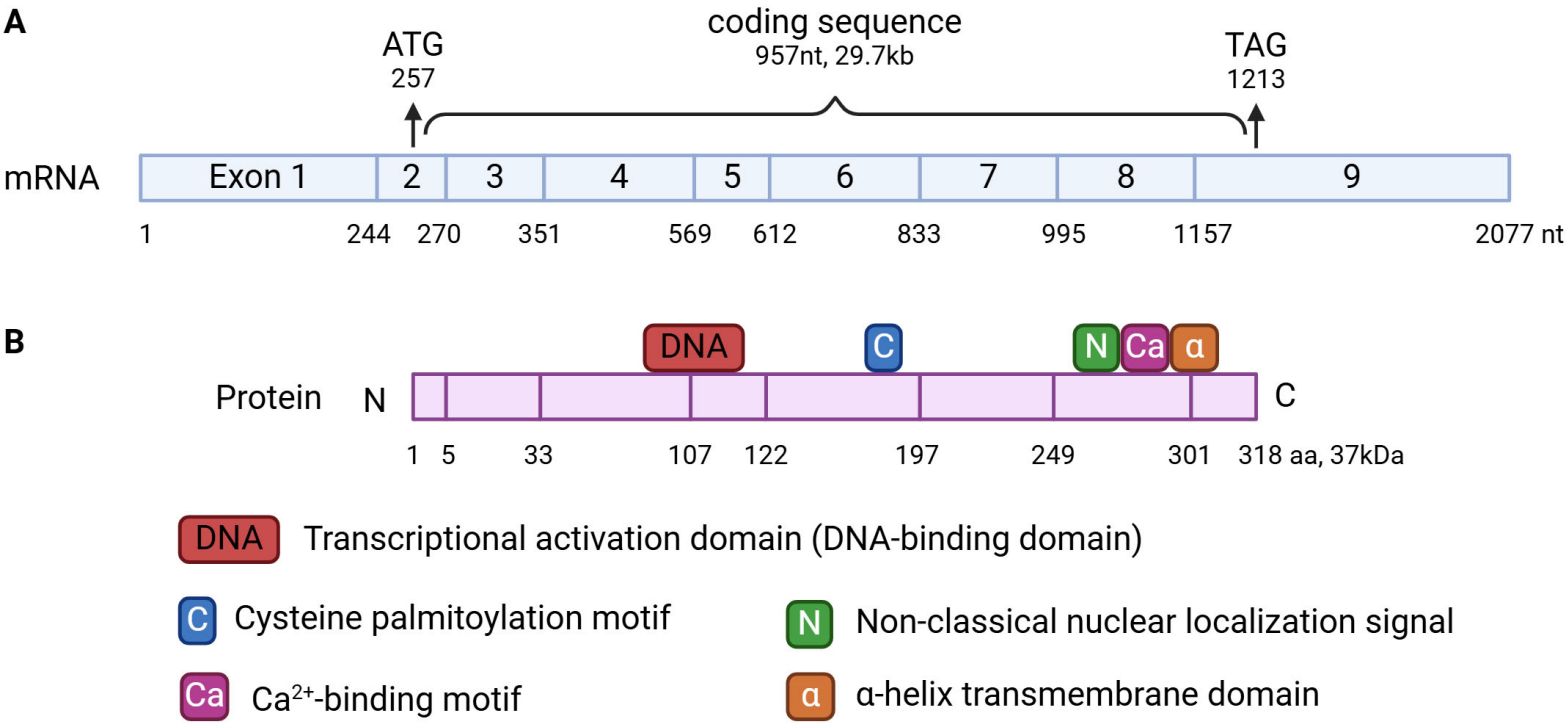
mRNA **(A)** and protein structure **(B)** of PLSCR1. **(A)** PLSCR1 mRNA is composed of 2077 nt with 9 exons. The 957nt-long coding region starts at 257nt in exon 2 and ends at 1213nt in exon 9. **(B)** The wildtype PLSCR1 protein is 318-aa-long. It possesses a single α-helical transmembrane domain (288-306 aa), a transcriptional activation domain (86-118 aa), a 5-cysteine palmitoylation motif (C^184^CCPCC^189^), a non-classical nuclear localization signal (NLS) (257-266 aa), and a Ca^2+^-binding motif (273-284 aa).

The phospholipid scramblase (PLSCR in human, Plscr in mice) family comprises 5 homologous proteins: PLSCR1-5. PLSCR1 is the first discovered and the most extensively studied member of the family. In 1996, PLSCR1 was first described by Basse et al. as an erythrocyte membrane protein that facilitates fibrin clotting by scrambling PE to the outer leaflet in response to Ca^2+^ influx (Basse et al., 1996). The full *PLSCR1* gene is composed of 2077 nucleotides (nt) with 9 exons (**Figure 1A**). The 957nt-long coding region starts at 257nt in exon 2 and ends at 1213nt in exon 9, producing a 318-amino acids (aa)-long wildtype PLSCR1 protein. As a type II transmembrane protein that possesses a single α-helical transmembrane domain (288-306 aa), PLSCR1 anchors to the membrane through its C-terminus, while its long extracellular N-terminus has all the functionally important domains: a transcriptional activation domain (86-118 aa), a 5-cysteine palmitoylation motif (C^184^CCPCC^189^), a non-classical nuclear localization signal (NLS) (257-266 aa), and a Ca^2+^-binding motif (273-284 aa) (**Figure 1B**) (Basse et al., 1996; Wiedmer et al., 2003; Chen et al., 2005; Bateman et al., 2009). Under normal conditions, PLSCR1 is localized on the plasma membrane. However, it can be internalized into endocytic vesicles or imported into the nucleus in response to stimuli such as epidermal growth factor (EGF), wogonoside, the cancer environment or influenza infections (Sun et al., 2002; Zhou et al., 2005; Wyles et al., 2007; Chen et al., 2013; Zhu et al., 2013; Luo, 2018; Huang et al., 2020; Yang et al., 2024). The cellular distribution of PLSCR1 is largely determined by its functional domains. For example, substitution of the cysteines in the cysteine palmitoylation motif with alanine completely abolishes PLSCR1’s membrane localization, leading to exclusive localization in the cytosol and nucleus (Wiedmer et al., 2003). On the other hand, a single amino acid mutation of histidine^262^ to tyrosine in the NLS of PLSCR1 completely disrupts its nuclear localization, leaving PLSCR1 exclusively in the cytosol and on the plasma membrane (Chen et al., 2005).

Although PLSCR1 was originally identified for its scramblase activity, it is actually a relatively weak and dispensable enzyme. Knockout of PLSCR1 *in vitro* did not affect the Ca^2+^-induced externalization of PS, while knockout TMEM16F, another human phospholipid scramblase, resulted in a profound defect in PS externalization (Xu et al., 2023). Moreover, overexpressing PLSCR1 only partially rescued PS externalization in TMEM16F knockout cells, suggesting that the scramblase activity of PLSCR1 can be compensated by other scramblases (Xu et al., 2023).

In fact, there are growing interests in investigating the transcriptional activation domain of PLSCR1, also known as its DNA binding domain. When imported into the nucleus, PLSCR1 often acts as a transcription factor to regulate gene expressions by directly binding to their promoter regions (Zhou et al., 2005; Chen et al., 2013; Huang et al., 2020; Yang et al., 2024), or by interacting with transcription factors to enhance its function (Zhu et al., 2013) (**Table 1**). For example, when stimulated with EGF, PLSCR1 not only directly binds to the promoter of *STAT1*, but also enhances STAT3’s binding to the *STAT1* promoter in breast cancer cells, leading to the transactivation of *STAT1* and basal-like breast cancer (BLBC) progression (Huang et al., 2020). Our groups’ recent publication discovered that in influenza A virus (IAV) infection, PLSCR1 binds to the promoter of IFN-λ receptor 1 (*IFN-λR1*) and enhances its transcription in ciliated airway epithelial cells, thus promoting viral clearance and reducing lung inflammation (Yang et al., 2024). Moreover, PLSCR1 binds to the promoter of inositol 1,4,5-triphosphate receptor type 1 (*IP3R1*) in mouse embryonic fibroblasts and kidney epithelial cells when stimulated with all-*trans*-retinoic acid (ATRA) (Zhou et al., 2005). In acute myeloid leukemia (AML), treatment with wogonoside, a Chinese herbal medicine, similarly induces PLSCR1 binding to *IP3R1*, resulting in AML cell cycle arrest and differentiation (Chen et al., 2013). Lastly, in addition to enhancing mRNA transcription, PLSCR1 also upregulates rRNA transcription by interacting with angiogenin (ANG), a known transcription factor of ribosomal DNA (rDNA) (Zhu et al., 2013). PLSCR1 acts as a co-transcription factor to promote ribosome biogenesis and cell proliferation. All of these findings have led researchers to explore whether PLSCR1 has major roles in other cellular processes beyond lipid distribution.

**Table 1:** PLSCR1 as an activating transcriptional factor.

Target Gene	Disease Condition	Stimulus	Cell Type	Result
*STAT1*	Basal-like breast cancer (BLBC)	Epidermal growth factor (EGF)	Breast cancer cells	Promoted BLBC progression
*IFN-λR1*	Influenza A virus (IAV) infection	IFN-λ	Ciliated airway epithelial cells	Promoted viral clearance and reduced lung inflammation
*IP3R1*	N/A	All-*trans*-retinoic acid (ATRA)	Murine embryonic fibroblasts and kidney epithelial cells	Regulation of cell proliferation and maturation
	Acute myeloid leukemia (AML)	Wogonoside	AML cells	Cell cycle arrest and differentiation
Ribosomal DNA (rDNA)	N/A	Angiogenin (ANG)	Yeast cells, HeLa cells	Enhanced rRNA transcription, ribosome biogenesis, and cell proliferation through interacting with ANG

### 1.3 PLSCR1 as an ISG

**Figure 2:**
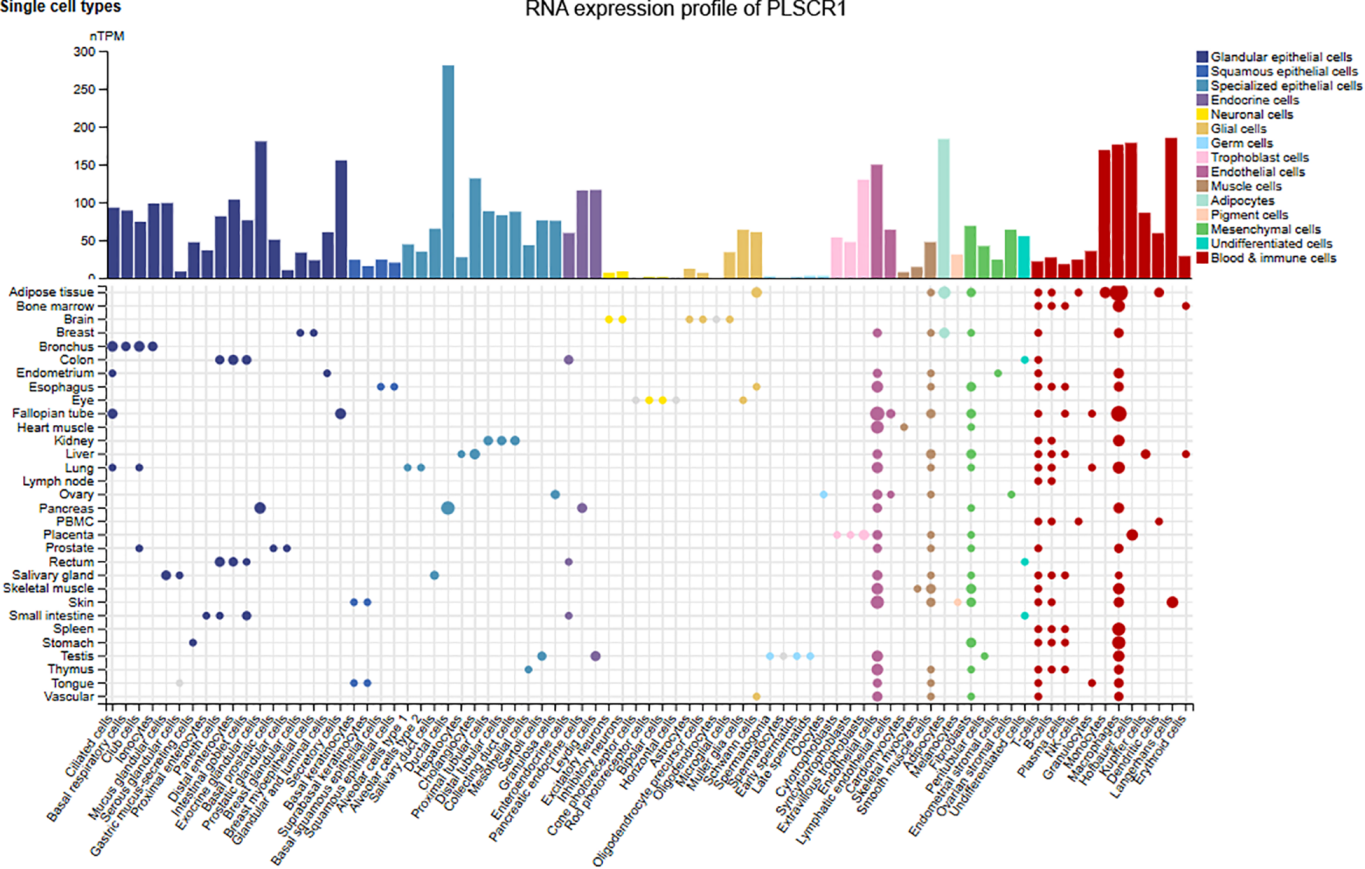
RNA expression profile of PLSCR1 across cell types in humans. PLSCR1 RNA expression was detected in all tissues with low tissue specificity in humans. Data were extracted from the Human Protein Atlas database.

PLSCR1 was identified as an antiviral ISG for the first time in 1998, as its expression can be highly induced by type 1 and 2 interferons (Der et al., 1998). According to the Human Protein Atlas database, PLSCR1 RNA is expressed by all tissues in humans with low tissue specificity at baseline (**Figure 2**) (Karlsson et al., 2021). The highest expression of PLSCR1 was observed in ductal cells in pancreas, while the lowest expressions were observed in neuronal cells of the eye and germ cells in the ovary and testes. Using oligonucleotide arrays in the human fibrosarcoma cell line HT1080, Der et al. demonstrated that PLSCR1 mRNA expression was increased 8-fold by IFN-α2a, 10-fold by IFN-β, and 3-fold by IFN-γ, making it one of the most highly inducible of novel ISGs identified. Later, a single IFN-stimulated response element (ISRE) located in the untranslated exon 1 of *PLSCR1* was identified as the regulator of the IFN-α2a-inducible transcription of *PLSCR1* in multiple human cell lines as well as primary cells (Zhou et al., 2000). The protein expression of PLSCR1 was increased up to 10 folds above basal level when stimulated with IFN-α2a *in vitro*. In hepatitis C virus (HCV) infection, PLSCR1 was upregulated by both IFN-α and IFN-γ, indicating its role as an ISG in HCV infections (Metz et al., 2012). Recent studies by others and our group further demonstrated that PLSCR1 is also a type 3 interferon-inducible gene in airway epithelial cells (Xu et al., 2023; Yang et al., 2024).

As of this review, a total of 16 research articles have reported the antiviral activities of PLSCR1 against 11 different species of viruses with diverse mechanisms (Dong et al., 2004; Kusano and Eizuru, 2012; Metz et al., 2012; Talukder et al., 2012; Yang et al., 2012; Kusano and Eizuru, 2013; Yuan et al., 2015; Luo, 2018; Kusano and Ikeda, 2019; Liu et al., 2022; Sadanari et al., 2022; Chen et al., 2023; Xu et al., 2023; Yang et al., 2024; Ma et al., 2025). Interestingly, there are 2 reports suggesting PLSCR1’s functions in promoting virus replication, and they will be briefly discussed in this review (Gong et al., 2011; Cheshenko et al., 2018). In addition, the roles of PLSCR1 in other cellular processes, such as cancer development, cell death, mast cell degranulation and more, have been reviewed in detail elsewhere and will not be discussed in this review (Kodigepalli et al., 2015; Dal Col et al., 2022). Based on these considerations, this review provides a comprehensive overview of the antiviral activities of PLSCR1 that have been published so far, with a particular focus on the underlying mechanisms.

## 2 Antiviral activities of PLSCR1 through interfering with viral replication

**Figure 3:**
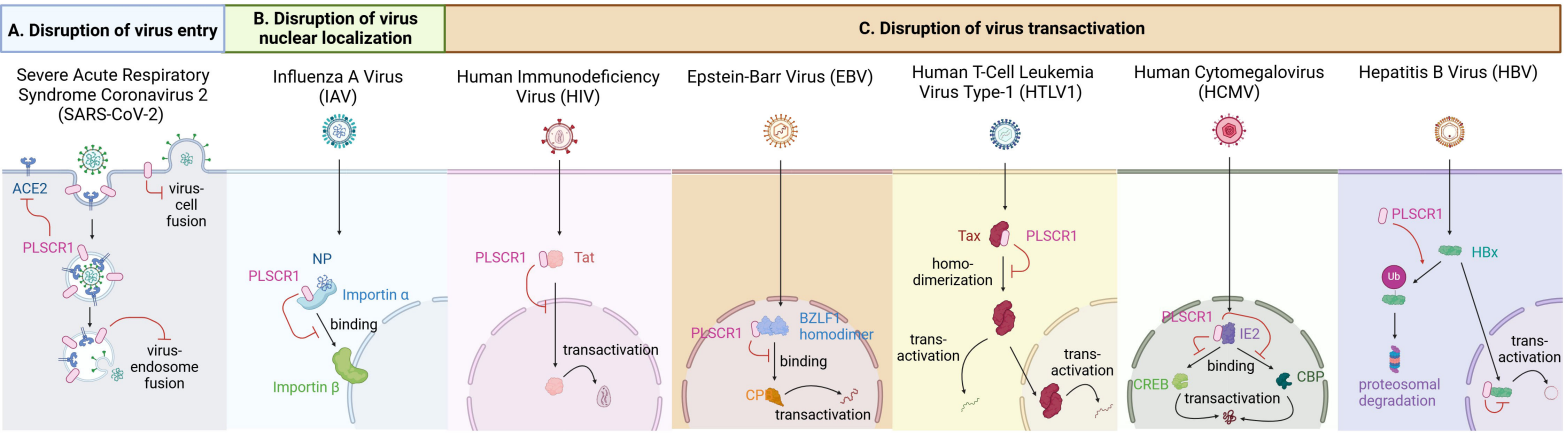
Antiviral activities of PLSCR1 through interfering with viral replication. **(A)** PLSCR1 disrupts virus entry of SARS-CoV-2 by inhibiting spike-mediated endosomal fusion, disrupting TMPRSS2-mediated cell-surface fusion, and downregulating cell surface ACE2. **(B)** PLSCR1 disrupts virus nuclear localization of IAV by interacting with NP and preventing PLSCR1-NP-importin α complex from binding importin β. **(C)** PLSCR1 disrupts virus transactivation of (1) HIV by interacting with Tat and reducing Tat nuclear localization, (2) EBV by decreasing transactivator complex BZLF1-CBP formation through direct binding to BZLF-1, (3) HTLV-1 by interacting with Tax and reducing its homodimerization, (4) HCMV by interacting with IE2, CREB and CBP and preventing their complex formation by direct binding competition, and (5) HBV by promoting HBx ubiquitination and proteasomal degradation through interaction in the nucleus.

A growing body of literature has now established that PLSCR1 is not only a lipid scramblase responsible for phospholipid redistribution, but also a critical anti-viral protein to defend against a large variety of viral species. Research has illustrated the ability of PLSCR1 to interact with viral antigens or virus-containing vesicles, whether on membrane, in cytoplasm or in nucleus. Here we describe the antiviral activities of PLSCR1 that directly interfere with one or more processes during viral replication, including virus entry, nuclear localization and gene expression (**Figure 3**, **Table 2**).

**Table 2:** Antiviral activities of PLSCR1 through interfering with viral replication.

Virus	Strain	PLSCR1’s Function	Mechanism	Study Model	Main Methods
SARS-CoV-2	• USA-WA1/2020 • Delta B.1.617.2 • Omicron B1.1.529	Disruption of virus entry	Inhibits both spike-mediated endosomal fusion and TMPRSS2-mediated cell-surface fusion	• Huh7.5 • A549-ACE2 • 293T-ACE2 • Calu-3 • Tonsil-ACE2 • HaCaT-ACE2 • HeLa-ACE2 • LET1-ACE2	• Genome-wide CRISPER-Cas9 screens • Whole-cell 4Pi single-molecule switching nanoscopy • Bipartite nano-reporter assays • AlphaFold2 prediction • PS externalization assay with ionomycin and FACS • Confocal IF • Luciferase assay
	• NY-RU-NY1 • Beta B.1.352 • Delta B.1.617.2 • Omicron BA.5 • Omicron XBB.1.5	Disruption of virus entry	Inhibits spike-mediated endosomal entry	• Huh7.5 • A549-ACE2 • 293T-ACE2 • Caco2 • Human SV40-fibroblasts-ACE2	• Genome-wide arrayed CRISPR screen • Confocal IF • Luciferase assay • Analysis of published large-scale omic studies
	Omicron	Disruption of virus entry	Downregulates cell surface ACE2	• A549-ACE2 • 293T-ACE2 • HeLa-ACE2 • Huh7.5.1	• Co-IP • Confocal IF • Luciferase assay • Flow cytometry • Cell fractionation
IAV	• A/Anhui/2/2005 (AH05, H5N1) • A/Anhui/1/2013 (AH13, H7N9) • A/WSN/1933 (WSN, H1N1) • A/Fuzhou/1/2009 (FZ09, H1N1)	Disruption of virus nuclear localization	Interacts with NP and prevents PLSCR1-NP-importin α complex from binding importin β	• HEK293T • A549 • THP-1 • U251	• Yeast two-hybrid screening • Co-IP • GST pull-down • Confocal IF • Cell fractionation
HIV	N/A	Disruption of virus transactivation	Interacts with Tat and reduces Tat nuclear localization	• COS-1 • MOLT/HIV	• Co-IP • Cell fractionation • Confocal IF • Luciferase assay
EBV	• EBV-1	Disruption of virus transactivation	Decreases transactivator complex BZLF1-CBP formation through direct binding to BZLF-1	• C666-1 • EBV-infected NPC xenograft C15 & C17 tumors • HEK-293 • COS-1 • HeLa-1 • A431 • MCF-7 • SW480 • BJAB • B95-8 • Namalwa • P3HR1 • Daudi	• Co-IP • Cell fractionation • Confocal IF • Luciferase assay
HTLV1	N/A	Disruption of virus transactivation	Interacts with Tax and reduces its homodimerization	• COS-1 • K3T	• Co-IP • Cell fractionation • Confocal IF • Luciferase assay
HCMV	• Towne • AD169	Disruption of virus transactivation	Interacts with IE2, CREB and CBP and prevents their complex formation by direct binding competition	• Human embryonic lung tissue-derived fibroblasts • OUMS-36T-3 • HEK-293	• Plaque assay • Co-IP • IF for subcellular localization • Luciferase assay
HBV	• N/A	Disruption of virus transactivation	Promotes HBx ubiquitination and proteasomal degradation through interaction in the nucleus	• HEK-293 • HepG2 • Huh7 • Human blood samples - Chronic HBV Carriers - HBV-hepatocellular carcinoma patients	• Yeast two-hybrid screening • Interactome analysis • Co-IP • GST pull-down • Ubiquitination assay • Cell proliferation assay • ELISA • IF

### 2.1 Plscr1 disrupts virus entry of severe acute respiratory syndrome coronavirus 2 (SARS-CoV-2)

As a virus responsible for the global pandemic, SARS-CoV-2 utilizes two entry mechanisms to invade host cells: spike protein-mediated endosomal fusion and TMPRSS2-mediated cell-surface fusion (Jackson et al., 2022). Xu et al. performed genome-wide CRISPR-Cas9 screening by transducing a GeCKO v2.0 single-guide RNA (sgRNA) library into human lung epithelial cells (Xu et al., 2023). After puromycin selection for stable knockout, transduced cells were pre-treated with IFN-γ and then infected with mNeonGreen (mNG)-labeled SARS-CoV-2. Using fluorescence-activated cell sorting (FACS) for mNG, infected cells were sorted based on their viral permissiveness, and their cellular DNA was extracted for next-generation sequencing of sgRNA frequencies. PLSCR1 was identified as a potent anti-SARS-CoV-2 defense factor due to its high enrichment score in the permissive mNG^high^ cell population, both with or without IFN-γ stimulation (Xu et al., 2023). At baseline, PLSCR1 is expressed abundantly by pulmonary epithelial cells including ciliated, club, and alveolar type 2 cells in humans (Karlsson et al., 2021), all of which express angiotensin-converting enzyme 2 (ACE2) and are the main targets of SARS-CoV-2 (Liu et al., 2021). Although the identify of intermediate hosts is still debated, SARS-CoV, MERS-CoV and SARS-CoV-2 are believed to have emerged through spillover from bats and are considered as bat-borne viruses (Irving et al., 2021). The anti-coronavirus property of PLSCR1 is evolutionarily conserved across viral variants of concern (VOCs), such as SARS-CoV-2 USA-WA1/2020, Delta B.1.617.2 and Omicron B1.1.529, and host species, such as humans, mice (experimental models) and bats (zoonotic ancestors) (Xu et al., 2023). With detailed examination of the viral replication cycle, Xu et al. discovered that PLSCR1 blocks coronavirus entry by specifically disrupting virus-membrane fusion, without affecting other steps like viral receptor expression, binding, externalization, trafficking or spike cleavage. In Huh7.5 and A549-ACE2 cells that naturally lack TMPRSS2, SARS-CoV-2 entry depends entirely on spike protein-mediated endosomal fusion, where cysteine proteases cleave the spike protein to expose a fusion peptide (Jackson et al., 2022). DMSO-treated PLSCR1^-/-^ Huh7.5 and A549-ACE2 cells lost protection against SARS-CoV-2, indicated by their high mNG intensity. The authors went on to use E-64d, a cysteine protease inhibitor, to block endosomal fusion. It was observed that E-64d treatment restored the protection against SARS-CoV-2 infection in *PLSCR1^-/-^* cells, indicating that PLSCR1 plays a critical role in SARS-CoV-2 entry via the endosomal pathway (Xu et al., 2023). On the other hand, Calu-3 cells express TMPRSS2, and E-64d treatment forced SARS-CoV-2 to rely on TMPRSS2-mediated cell-surface fusion. Further inhibition of TMPRSS2 with camostat significantly reduced the susceptibility of *PLSCR1^-/-^* Calu-3 cells against SARS-CoV-2 infection, indicating that PLSCR1 also restricts SARS-CoV-2 entry via the cell-surface fusion mechanism. Additionally, both the subcellular location and structural integrity of PLSCR1 are essential for its anti-coronavirus activities: PLSCR1 must localize on the cell membrane and maintain its β-barrel conformation to effectively inhibit viral entry. The H262Y mutation of PLSCR1 affects the β-barrel conformation of the protein, thus impairing its anti-SARS-CoV-2 activity. However, the enzymatic activity of PLSCR1 does not appear to be involved in its anti-SARS-CoV-2 activity.

Le Pan et al. confirmed the findings of Xu et al., demonstrating PLSCR1 as an intrinsic barrier to SARS-CoV-2 entry (Le Pen et al., 2024). In their genome-wide arrayed CRISPR screen, PLSCR1 emerged as one of the most potent anti-SARS-CoV-2 genes at baseline, with its effect further enhanced by IFN-α2a pretreatment, which increased PLSCR1 expression levels. Notably, inhibition of type 1 IFN signaling with a JAK-STAT inhibitor did not affect PLSCR1’s ability to limit SARS-CoV-2 infection. In addition, loss of PLSCR1 did not impair type 1 IFN signaling during SARS-CoV-2 infection, as evidenced by unaltered expressions of two ISGs, *OAS1* and *IF16*. This suggests that PLSCR1 has a cell-intrinsic anti-SARS-CoV-2 mechanism independent of IFN pathway. However, a broader examination of ISGs, particularly those previously identified as PLSCR1-dependent (Dong et al., 2004), may be necessary to strengthen this conclusion. Consistent with prior findings (Xu et al., 2023), Le Pan et al. demonstrated that PLSCR1 did not restrict a SARS-CoV-2 replicon system that bypasses cell entry, but effectively controlled replication-defective, spike-coated SARS-CoV-2 pseudoviruses in Huh7.5 cells, highlighting its role in blocking spike-mediated endosomal entry (Le Pen et al., 2024). PLSCR1 was also shown to restrict viral variants, including Beta B.1.352, Delta B.1.617.2, Omicron BA.5 and Omicron XBB.1.5. However, its restriction of newer strains was less efficient in Huh7.5 cells, suggesting potential viral adaptation to antagonize PLSCR1. Lastly, Le Pan et al. also investigated the H262Y mutation in PLSCR1, and determined that it had a dominant-negative effect on anti-SARS-CoV-2 protection in transfected A549-ACE2 cells and heterozygous human SV40-fibroblast-ACE2 cells, corroborating the findings of Xu et al. (2023).

More recently, another mechanistic study revealed that PLSCR1 inhibits SARS-CoV-2 entry by specifically downregulating cell surface ACE2 expression, while leaving the overall cellular expression of ACE2 unchanged (Ma et al., 2025). In a smaller screen of 109 ISG-knockout ACE2-overexpressing A549 cell lines, created by CRISPR-Cas9 editing, Ma et al. identified PLSCR1 as the most potent anti-SARS-CoV-2 ISG. Consistent with previous reports (Xu et al., 2023; Le Pen et al., 2024), they found that PLSCR1 inhibits SARS-CoV-2 replication by blocking spike-mediated viral entry. Specifically, spike-coated pseudovirus entered PLSCR1^-/-^ A549-ACE2 cells more efficiently and promoted spike and ACE2-mediated cell-cell fusion (Ma et al., 2025). Furthermore, PLSCR1 demonstrated broad-spectrum inhibitory effects against the entry of pseudoviruses bearing spike protein from multiple SARS-CoV-2 variants, including Wuhan, Alpha, Beta, Gamma, Delta and Omicron (Ma et al., 2025). Additionally, treatment of R5421, a pharmacological inhibitor of PLSCR1’s scramblase activity, did not affect the anti-SARS-CoV-2 activity of PLSCR1, further supporting Xu et al.’s findings (Xu et al., 2023). Noteworthily, a series of negative co-immunoprecipitation (Co-IP) results confirmed that PLSCR1 does not physically interact with either spike protein or its subunits (S1, S2 and RBD), nor with host cell receptors (ACE2 and TMPRSS2) (Ma et al., 2025). Instead, flow cytometry and cell fractionation assay showed that PLSCR1 inhibits surface localization of ACE2 without altering its total expression at baseline. Finally, Ma et al. showed that the H262Y mutation of PLSCR1 partially ablates its inhibitory effect on SARS-CoV-2 entry, as the H262Y mutant is unable to downregulate cell surface ACE2 expression. While this proposed mechanism provides a plausible explanation for the effect of H262Y mutation observed in all 3 studies (Xu et al., 2023; Le Pen et al., 2024; Ma et al., 2025), further molecular studies are needed to fully elucidate how this single amino acid substitution in PLSCR1 impacts its regulatory role.

### 2.2 Plscr1 disrupts virus nuclear localization of IAV

PLSCR1 was shown to physically interact with the viral nucleoprotein (NP) of IAV, impairing its nuclear import and thereby suppressing virus replication *in vitro* (Luo, 2018). As a single-stranded, negative-sense RNA virus that transcribes and replicates in the nucleus of host cells, IAV relies on NP for nuclear import of the viral ribonucleoprotein complex (vRNP) (O’Neill et al., 1995). Specifically, NP binds to the host heterodimeric importin α/β complex in the cytoplasm and enters the nucleus through the classical nuclear import pathway (Wang et al., 1997). Luo et al. identified PLSCR1 as a binding partner of NP using yeast two-hybrid screening, Co-IP and GST pull-down assays in A549, HEK293T, THP-1 and/or U251 cells (Luo, 2018). When bound by PLSCR1 in the cytoplasm, the PLSCR1-NP-importin α complex is prevented from binding importin β, thus suppressing the classical nuclear import pathway. Noteworthily, overexpression of PLSCR1 efficiently inhibited nuclear import of NP and negatively regulated IAV replication, as indicated by reduced viral titers and RNA levels. Conversely, treatment of IAV-infected A549 and human bronchial epithelial BEAS-2B cells with the PLSCR1 inhibitor R5421 dose-dependently increased viral titers (Wiwie et al., 2019). However, intrinsic cellular factors could interfere with this mechanism. Liu et al. were the first to demonstrate the anti-influenza effects of PLSCR1 in a mouse model. They found that immunoglobulin-like domain-containing receptor 1 (ILDR1) competes with NP for binding to PLSCR1 in H1N1 swine influenza virus (SIV) infection (Liu et al., 2022). As a type 1 transmembrane protein highly expressed in the lungs following infection, ILDR1 promotes SIV replication by inhibiting the PLSCR1-NP interaction. Therefore, ILDR1 inhibitors may enhance PLSCR1’s anti-influenza activities.

### 2.3 Plscr1 disrupts virus transactivation

From 2012 to 2022, the Kusano group have reported the ability of PLSCR1 to disrupt transactivation of multiple viruses, including human immunodeficiency virus (HIV), Epstein-Barr virus (EBV), human T-cell leukemia virus type-1 (HTLV1) and human cytomegalovirus (HCMV) (Kusano and Eizuru, 2012; Kusano and Eizuru, 2013; Kusano and Ikeda, 2019; Sadanari et al., 2022). Disruption of hepatitis B virus (HBV) transactivation was also documented by Yuan and colleagues (Yuan et al., 2015). Here we aim to compare and contrast the underlying mechanisms regulated by PLSCR1.

#### 2.3.1 HIV

PLSCR1’s interaction with HIV Tat (trans-activator of transcription) has been reported to inhibit HIV transactivation by reducing Tat nuclear localization (Kusano and Eizuru, 2013). Tat is a small transactivator protein encoded by HIV-1 that is essential for transcription of provirus and replication of HIV-1 (Gautier et al., 2005). Upon translocation to the nucleus, Tat recruits positive transcription elongation factor b (P-TEFb), which phosphorylates RNA polymerase II and activates transcription from the HIV-1 long terminal repeat (LTR) (Zhou and Yik, 2006). Using Co-IP, Kusano and Eizuru demonstrated that both a PLSCR1 plasmid construct and IFN-induced endogenous PLSCR1 expressed by COS-1 cells directly interact with Tat (Kusano and Eizuru, 2013). Specifically, amino acids 160-250 of PLSCR1 contain 2 binding sites for Tat with different affinities: the weaker site is located within amino acids 200-250, and the stronger site is located within amino acids 160-205. Full-length PLSCR1, but not truncated PLSCR1(160-250), significantly represses Tat-mediated HIV-1 transactivation, suggesting that the favorable binding site within amino acids 200-250 is required for anti-HIV activities. In addition, in the presence of PLSCR1(160-250) or the absence of PLSCR1, Tat was primarily detected in the nucleus of COS-1 cells. However, in the presence of full-length PLSCR1, Tat was observed throughout both the cytoplasm and nucleus, and it colocalized with PLSCR1 in the cytoplasm. Consistently, cell fractionation assay showed an increased fraction of cytoplasmic, stabilized Tat with full-length PLSCR1 expression. Therefore, PLSCR1-Tat interaction blocks Tat from nuclear localization, thereby repressing Tat-dependent transactivation of HIV. In human immune cell populations, PLSCR1 is highly expressed in monocytes and macrophages, while its expression is lower in T cells (Karlsson et al., 2021). All of these cell types are susceptible to HIV infection (Wong et al., 2019). Recently, PLSCR1 was identified as a significant monocyte marker by single-cell transcriptome sequencing of peripheral blood mononuclear cells (PBMCs) associated with HIV replication, indicating that PLSCR1-Tat interaction may play a critical role in immunological non-responsiveness in monocytes (Chen et al., 2023).

#### 2.3.2 EBV

In another study by Kusano and Ikeda, PLSCR1 was implicated in EBV infection, as it interacts with EBV protein BZLF1 and represses BZLF1-mediated lytic gene transcription in EBV-infected nasopharyngeal carcinoma (NPC) (Kusano and Ikeda, 2019). EBV is a major cause of NPC and often establishes lytic infections in squamous epithelial cells but latent infections in B cells (Raab-Traub, 2002). BZLF1 is an EBV-encoded immediate-early lytic transactivator that initiates the transcription of early genes for viral DNA replication (Tsurumi et al., 2005). Basal PLSCR1 expression is highly induced in EBV-infected NPC cell line C666-1 and xenograft tumors C15 and C17, but is undetectable in EBV-positive B cells, suggesting its role in the switch from latent to lytic EBV infections (Kusano and Ikeda, 2019). While baseline PLSCR1 expressions is low in both human noncancer squamous epithelial cells and B cells (Karlsson et al., 2021), the observations from this study suggest that EBV specifically induces PLSCR1 expression in squamous epithelial cells, but not in B cells (Kusano and Ikeda, 2019). Co-IP results demonstrated a direct interaction between PLSCR1 and BZLF1 and identified two BZLF1-binding sites on PLSCR1 (amino acids 1-163 and 160-250). Correspondingly, amino acids 170-196 of BZLF1, located within the C-terminal DNA-binding domain of the bZIP motif, are required for interaction with PLSCR1. Unlike HIV Tat (Kusano and Eizuru, 2013), EBV BZLF1’s nuclear localization was not affected by PLSCR1 binding (Kusano and Ikeda, 2019). However, in a similar way, BZLF1-mediated transactivation was efficiently repressed by PLSCR1, especially through the amino acids 1-163 fragment, in a dose-dependent manner. The transcription of BMRF1, an EBV early and lytic gene, was significantly reduced when PLSCR1 was overexpressed in C666-1 cells. To transactivate EBV early genes such as BMRF1, BZLF1 needs to homodimerize and interact with the transcription co-activator CBP through its bZIP region (Adamson and Kenney, 1999). While PLSCR1 does not affect BZLF1 homodimerization, it outcompetes CPB for binding to the BZLF-1 bZIP domain (Kusano and Ikeda, 2019). Taken together, PLSCR1 disrupts EBV lytic gene expressions by decreasing the formation of its transactivator complex, BZLF1-CBP, through direct binding competition.

#### 2.3.3 HTLV-1

PLSCR1’s role in regulating HTLV-1 transactivation was also documented by Kusano and Eizuru (2012). The homodimerized HTLV-1 transactivator Tax is responsible for efficient transcription of HTLV-1 provirus, which could lead to T-cell leukemia, lymphoma and neurodegenerative diseases in adults (Yoshida et al., 1982; Jin and Jeang, 1997). Baseline PLSCR1 expressions is low in human T cells and non-Tax-producing HTLV-1-infected adult T-cell leukemia (ATL) cell line (Kusano and Eizuru, 2012; Karlsson et al., 2021). However, PLSCR1 expression was remarkably induced by treatment of IFN-α2b in ATL cell line (Kusano and Eizuru, 2012). Co-IP results demonstrated a direct interaction between PLSCR1 and Tax, identifying amino acid 2-115 on Tax as a single binding site for PLSCR1 (Kusano and Eizuru, 2012). Correspondingly, PLSCR1 has two binding sites for Tax: one within amino acids 1-100, and the other within amino acid 160-250. In HTLV-1-infected and Tax-transfected cells, Tax distributes throughout both the cytoplasm and nucleus (Tsuji et al., 2007). The primary role of Tax in the cytoplasm is to transactivate several cellular pathways, including nuclear factor (NF)-κB through protein-protein interactions (Boxus et al., 2008). Surprisingly, PLSCR1 significantly reduces the cytoplasmic localization of Tax and co-localizes with Tax in the nucleus (Kusano and Eizuru, 2012). Further examination revealed that Tax-mediated transactivation of NF-κB-regulated reporter constructs was indeed inhibited by PLSCR1 in K3T cells (a tax-producing HTLV-1-infected T cell line). On the other hand, Tax in the nucleus facilitates the transactivation of 5’ long terminal repeat (LTR) of HTLV-1 (Jin and Jeang, 1997). Similar to HIV Tat and EBV BZLF1 (Kusano and Eizuru, 2013; Kusano and Ikeda, 2019), HTLV-1 Tax-mediated transactivation of HTLV-1 5’ LTR was efficiently repressed by PLSCR1 in K3T cells (Kusano and Eizuru, 2012). Interestingly, Kusano and Eizuru found that endogenous PLSCR1 expression in K3T cells was very low at baseline and barely upregulated by type 1 IFN stimulation. Since Tax has been previously reported to inhibit the IFN signaling pathway in T cells (Charoenthongtrakul et al., 2011), ISG expression, including PLSCR1, may be repressed as a countermeasure by HTLV-1. Finally, PLSCR1 also reduces the homodimerization of Tax *in vitro*. In conclusion, PLSCR1 disrupts transactivation of both NF-κB-regulated reporter constructs in the cytoplasm and HTLV-1 5’ LTR in the nucleus through its interaction with HTLV-1 Tax.

#### 2.3.4 HCMV

More recently, Sadanari et al. from the Kusano group discovered that PLSCR1 restricts the transactivation of HCMV (Sadanari et al., 2022), a herpesvirus that is usually asymptomatic in healthy individuals but can cause severe diseases in infants and immunocompromised patients (Sissons and Carmichael, 2002). HCMV infects fibroblasts, endothelial, and epithelial cells by suppressing host IFN signaling, and IFN pretreatment has been shown to inhibit HCMV replication (Sainz et al., 2005; Gerna et al., 2019). Hence, ISGs such as PLSCR1, which have relatively high basal expressions in HCMV-permissive fibroblasts, endothelial and epithelial cells, and can be further induced by IFNs, may play a role in the anti-HCMV defense. Sadanari et al. observed that when infected with HCMV, 36T-3 cells, an immortalized human embryo fibroblast cell line with higher basal PLSCR1 expression, produced fewer plaques and expressed fewer major immediate early (MIE) and early proteins compared to HEL cells, which are human embryonic lung tissue-derived fibroblasts with lower basal PLSCR1 expression (Sadanari et al., 2022). However, 36T-3 cells lost their resistance to HCMV when PLSCR1 was knocked-out, demonstrating that endogenous PLSCR1 inhibits HCMV replication by suppressing MIE and early gene expressions. The HCMV immediate early protein 2 (IE2)-CREB and IE2-CBP complexes are essential for transactivation of early gene promoters (Schwartz et al., 1996). Co-IP analysis showed that PLSCR1 interacts with IE2, CREB and CBP, preventing their complex formation by direct binding competition (Sadanari et al., 2022). As a result, CREB-mediated transcription from the HCMV MIE protein promoter is disrupted by PLSCR1.

#### 2.3.5 HBV

Despite vaccination efforts, HBV infection remains a major etiological cause of cirrhosis and/or hepatocellular carcinoma (HCC) worldwide (Dienstag, 2008). HBV X (HBx) is a transactivator that regulates many viral and cellular processes, including transcription, signaling, cell cycle progression, cell death, protein degradation and genetic stability (Motavaf et al., 2013). Through yeast two-hybrid screening of a human liver cDNA library, Yuan et al. identified PLSCR1 as an HBx-binding partner that suppresses HBx protein expression (Yuan et al., 2015). However, treatment with proteasome inhibitor MG132 erased this effect, indicating that PLSCR1 promotes HBx degradation in the proteasome. Consistently, ubiquitination of HBx, an initial step in proteasomal degradation, was increased in the presence of PLSCR1 and MG132. Furthermore, HBx is known to transactivate cell proliferation pathways associated with oncogenesis (Gong et al., 2013). PLSCR1 overexpression successfully repressed HBx-induced proliferation in HepG2 cells, suggesting its potential role in anticarcinogenesis defense against HBV (Yuan et al., 2015). Additional Co-IP analysis showed that PLSCR1 physically binds HBx in the nucleus. When the nuclear localization of PLSCR1 was disrupted, the binding affinity was significantly reduced, and no inhibition of HBx expression was observed. In addition to *in vitro* experiments, Yuan et al. also examined clinical human samples. While healthy human hepatocytes at baseline express a low level of PLSCR1 (Karlsson et al., 2021), they found that PLSCR1 levels were significantly higher in the plasma of chronic HBV carriers (CHB) compared to HCC patients and healthy controls, implying that CHB patients with lower PLSCR1 levels may be at higher risk of developing HCC (Yuan et al., 2015). In conclusion, PLSCR1 promotes the ubiquitination and proteasomal degradation of HBx, disrupting HBx-mediated transactivation of cell proliferation *in vitro*. However, *in vivo* experiments are needed in the future to confirm that PLSCR1 plays a definitive role in suppressing HBV infection and carcinogenesis at tissue and organismal level.

## 3 Antiviral activities of PLSCR1 through regulation of immune components

**Figure 4:**
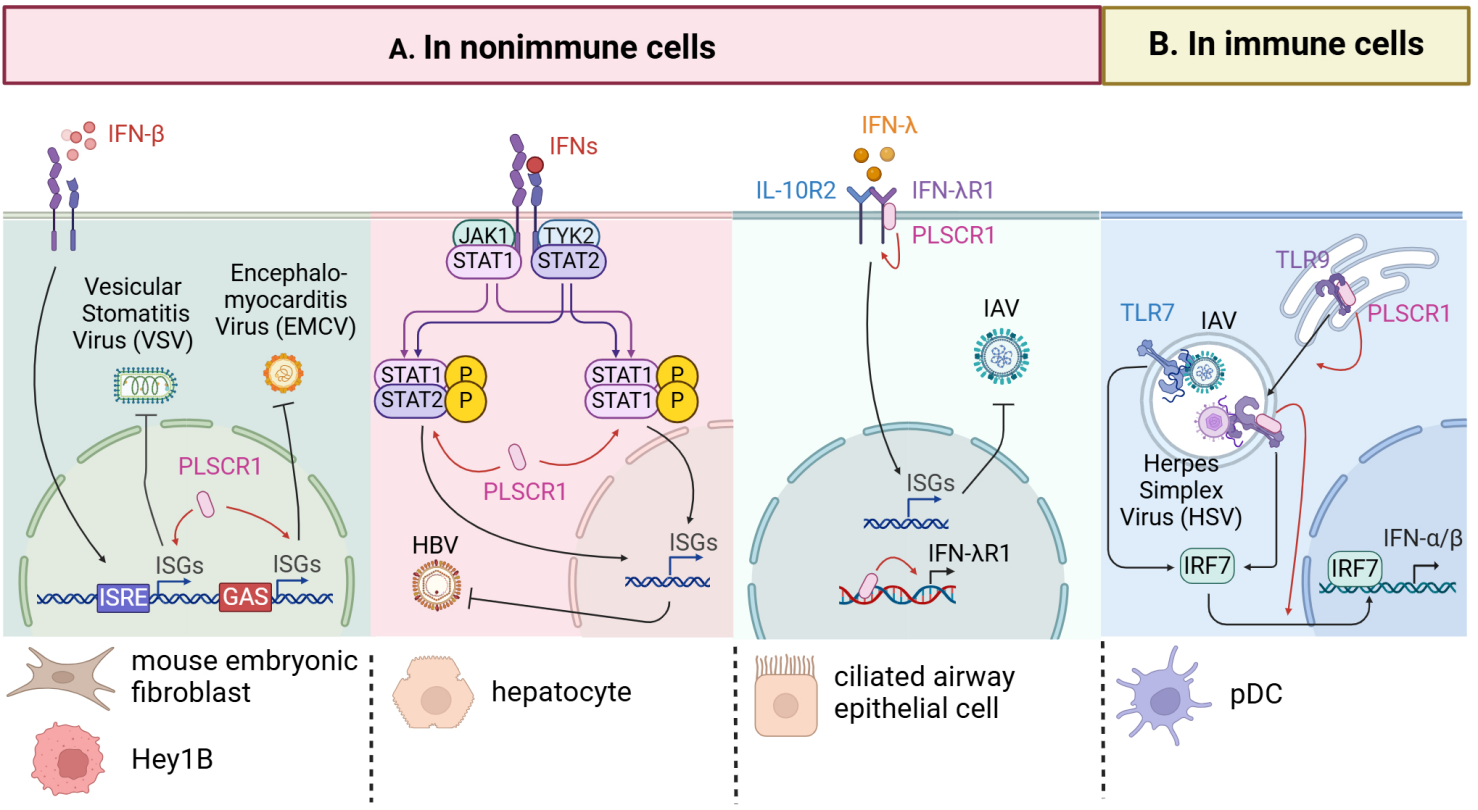
Antiviral activities of PLSCR1 through regulation of immune components. **(A)** PLSCR1 mediates immune regulations in nonimmune cells. In VSV and EMCV infections, PLSCR1 potentiates the expressions of a subset of ISGs in mouse embryonic fibroblasts and breast carcinoma cells. In HBV infection, PLSCR1 induces the expression of phosphorylated STAT1 and STAT2 in hepatocytes. In IAV infection, PLSCR1 binds IFN-λR1 DNA and protein thus promoting IFN-λR1 expression in ciliated airway epithelial cells. **(B)** PLSCR1 mediates immune regulations in immune cells. In IAV and HSV-1 infections, PLSCR1 interacts with TLR9, regulates its trafficking from ER to endosome, and induces type I interferon production by TLR7 and TLR9 in pDCs.

In addition to directly controlling the viral replication cycle, PLSCR1 regulates host immune responses that contribute to virus clearance. As a widely expressed ISG, PLSCR1 interacts with inflammatory proteins, cell-death related proteins, and membrane receptors to modulate host hemostasis in infections. Moreover, our recent studies highlighted the importance of PLSCR1’s interaction with endogenous DNA and its role in transcriptional regulation as key mechanisms in antiviral defense. In this section, we will discuss the documented antiviral functions of PLSCR1 in regulating host immune components in both immune and nonimmune cell populations (**Figure 4**, **Table 3**).

**Table 3:** Antiviral activities of PLSCR1 through regulation of immune components.

Virus	Strain	PLSCR1’s Function	Mechanism	Study Model	Main Methods
VSV	• VSV-IND	Immune regulation in mouse embryonic fibroblasts	Potentiates the expressions of a subset of ISGs	• Mouse embryonic fibroblasts • Hey1B	• cDNA microarray • Plaque assay • Immunoblot
EMCV	N/A				
HBV	• Genotype D	Immune regulation in hepatocytes	Induces the expression of phosphorylated STAT1 and STAT2	• HepG2.2.15 • HepG2 • Huh7 • Balb/c mice	• ELISA • MTS assay • Hydrodynamics-based transfection in mice • IHC
IAV	• A/WSN/1933 (WSN, H1N1) • A/PR/8/1934 (PR8, H1N1)	Immune regulation in ciliated airway epithelial cells	Binds IFN-λR1 DNA and protein thus promoting IFN-λR1 expression	• C57BL/6J mice *- Plscr1^-/-^* *- Plscr1^floxStop^LysM-Cre^+^* *- Plscr1^floxStop^Foxj1-Cre^+^* • Calu-3 • A549	• BAL cytospin and Diff-Quik stain • H&E staining • IF • RNA sequencing (bulk & single-cell) • CHIP • PLA • Co-IP • Plaque assay
IAV, HSV-1	N/A	Immune regulation in pDCs	Interacts with TLR9, regulates its trafficking from ER to endosome, and induces type I interferon production by TLR7 and TLR9	• BM-derived *Plscr1^-/-^* & *Wt* mouse pDCs • Monocyte-derived *Plscr1^-/-^* & *Wt* mouse DCs • GEN2.2 • HEK-293T	• Yeast two-hybrid screening • Co-IP • ELISA • Confocal IF

### 3.1 Plscr1 regulates immune responses in nonimmune cells

#### 3.1.1 Fibroblasts and ovarian carcinoma cells

In a collaborative study by the Sims and Silverman groups, Dong et al. demonstrated that PLSCR1 inhibits infections by vesicular stomatitis virus (VSV) and encephalomyocarditis virus (EMCV) in both the human ovarian carcinoma cell line Hey1B and mouse embryonic fibroblasts. This inhibition occurs through potentiating the transcription of a subset of ISGs (Dong et al., 2004). When treated with IFN-β, a robust ISG p56 response was induced in Hey1B cells, leading to repressed VSV N protein production. A similar reduction in viral infection was observed in EMCV-infected cells, as EMCV viral titer was reduced with IFN-β pretreatment. However, these antiviral effects were abolished when PLSCR1 was knocked down, suggesting PLSCR1 may contribute to the expression of ISGs. Dong et al. also showed that PLSCR1 overexpression significantly reduced VSV titer, RNA transcripts and protein accumulation in mouse embryonic fibroblasts, without affecting viral absorption, penetration and budding. Furthermore, DNA microarray analysis showed that, compared to WT Hey1B cells, PLSCR1 knocked-down led to reduced expression of 24 ISGs. The ISGs that failed to be upregulated in the absence of PLSCR1 included ISG54, p56, ISG15 and STAT1, while PKR and RNase L were unaffected, suggesting that PLSCR1 specifically regulates a subset of ISGs. However, it remains unclear whether PLSCR1 regulates ISG gene transcription in the nucleus, or regulates the JAK/STAT signaling pathway on the membrane. Further studies are needed to explore the underlying mechanism of PLSCR1’s regulation on ISG expression in fibroblasts and ovarian carcinoma cells.

#### 3.1.2 Hepatocytes

As discussed in the previous section, PLSCR1 disrupts HBV transactivation through its interaction with viral transactivator HBx (Yuan et al., 2015). In addition, Yang et al. described that PLSCR1 activated the JAK/STAT signaling pathway following HBV infection (Yang et al., 2012). Similar to the downregulation of PLSCR1 in HTLV-1-infected T cells (Kusano and Eizuru, 2012), both PLSCR1 protein and mRNA levels were downregulated in a time-dependent manner in HBV1.3-transfected HepG2 cells, likely as a countermeasure by HBV (Yang et al., 2012). However, when PLSCR1 was transfected into these cells, it significantly inhibited the intracytoplasmic production of HBV surface antigens (HBsAg and HBeAg) in a dose-dependent manner. This effect was also observed in an acute HBV infection mouse model using hydrodynamic-based transfections. Moreover, PLSCR1 reduced HBV mRNA levels and core-associated viral DNA in HepG2 cells, consistent with the disruption of transactivation (Yuan et al., 2015). The JAK/STAT pathway, which is activated downstream of all interferon receptors, plays a pivotal role in the activation of transcription factors and the expression of ISGs during viral infections (Hu et al., 2021). Importantly, PLSCR1 promotes the phosphorylation of STAT1 and STAT2 in HBV-transfected HepG2 cells, indicating that the observed anti-HBV activities may be, at least partially, mediated by PLSCR1-induced JAK/STAT activation in hepatocytes (Yang et al., 2012). However, further studies are needed to investigate whether PLSCR1-mediated JAK/STAT signaling affects the expressions of downstream antiviral genes in HBV-infected hepatocytes.

Similar anti-viral mechanisms of PLSCR1 was identified in hepatocytes in the inhibition of HCV (Metz et al., 2012). In this study, an IFN-treated subgenomic HCV luciferase reporter replicon cell line (LucUbiNeoET) was used for an siRNA-based assay to knock down individual ISG candidates and screen for those that could rescue HCV replication. PLSCR1 was one of the top hits, and its expression was upregulated in response to HCV infection in primary human hepatocytes. Consistently, overexpression of PLSCR1 in Huh7 cells reduced HCV replication by ~50%, although the exact cellular mechanisms remained unclear. However, another study suggested that membrane-bound PLSCR1 could facilitate HCV attachment and entry by interacting with HCV envelop proteins E1 and E2 on hepatocytes (Gong et al., 2011). This will be discussed in a later section of this review.

#### 3.1.3 Lung epithelial cells

Most recently, our group identified that PLSCR1 enhances anti-IAV responses by promoting type 3 IFN (IFN-λ) signaling in ciliated airway epithelial cells (Yang et al., 2024). Our results showed that Plscr1 expression was significantly induced by IAV infection *in vivo* and in airway epithelial cells treated with IFN-λ. We found that *Plscr1^-/-^* mice exhibited exacerbated body weight loss, decreased survival rates, increased viral replication, and more severe lung damage. Notably, RNA sequencing analyses demonstrated that *Plscr1^-/-^* mice failed to upregulate their *Ifn-λr1* expression, along with the expression of a large subset of ISGs that are likely downstream of type 3 IFNs, upon IAV infection. The impaired expression of Ifn-λr1 and these downstream ISGs may contribute to delayed viral clearance in *Plscr1^-/-^* mice. Using chromatin immunoprecipitation (CHIP), we identified PLSCR1 as a transcriptional activator of *IFN-λR1*, as it directly binds to the promotor of *IFN-λR1* in IAV infection. In addition, PLSCR1 was found to interact with IFN-λR1 on the cell membrane of pulmonary epithelial cells following IAV infection, suggesting that PLSCR1 may modulate IFN-λ signaling via protein-protein interactions.

Similar to the work by Xu et al. (2023), we also uncoupled the lipid scramblase activity of Plscr1 from its anti-flu activity (Yang et al., 2024), suggesting that the scramblase activity of PLSCR1 may not be essential for its antiviral activities against multiple viruses. We also studied the H262Y mutation of PLSCR1 and found that it offers partial protection against IAV infection. While H262Y was found to be partially protective against SARS-CoV-2 infection due to its inability to downregulate ACE2 surface expression (Ma et al., 2025), the mechanism underlying its partial protection in IAV infection is due to its loss of nuclear localization and inability to function as a transcription factor for *IFN-λR1* (Yang et al., 2024).

Ciliated epithelial cells in the bronchus and lungs express PLSCR1 abundantly at baseline in humans (Karlsson et al., 2021). Noticeably, IAV predominately infects ciliated cells in humans, and the tropism for these cells correlates with viral burst size (Roach et al., 2024). Moreover, ciliated epithelial cells are the only cell type in the mouse airway that express α2,3-linked SA, the primary receptor for influenza virus (Ibricevic et al., 2006). Our single-cell RNA sequencing data indicated that *Plscr1* expression was significantly upregulated in ciliated airway epithelial cells in mice following IAV infection. Consistently, *Plscr1^floxStop^Foxj1-Cre^+^* mice, in which Plscr1 was specifically overexpressed in ciliated airway epithelial cells, exhibited reduced susceptibility, less inflammation, and enhanced Ifn-λr1 expression during IAV infection, indicating that Plscr1 mainly regulates type 3 IFN signaling as a cell intrinsic defense factor against IAV in ciliated airway epithelial cells. These findings suggest that targeting PLSCR1 to develop novel anti-influenza therapeutics may mitigate the emergence of drug-resistant IAV strains.

### 3.2 PLSCR1 regulates immune responses in dendritic cells

PLSCR1 interacts with Toll-like receptor 9 (TLR9) and regulates its ability to induce type I IFN production in response to CpG-ODN (oligodeoxynucleotide), herpes simplex virus (HSV) and IAV in plasmacytoid dendritic cells (pDCs) (Talukder et al., 2012). Dendritic cells exhibit moderate to high levels of PLSCR1 expression compared to other cell types in humans (Karlsson et al., 2021). TLR9 is a pathogen recognition receptor (PRR) that specializes in sensing microbial DNA, especially unmethylated CpGs of viral origin (Ewald et al., 2008). TLR9 signaling leads to the phosphorylation and nuclear import of IFN regulatory factor 7 (IRF7) and activation of type I IFNs in pDCs, which are key producers of type I IFN in the immune system (Cella et al., 1999; Honda et al., 2005). As reported by Talukder et al., PLSCR1 was identified as a binding partner of TLR9 by yeast two-hybrid screening, and endogenously expressed TLR9 interacted with the N-terminal G-box domain of PLSCR1 in human pDC cell line (Talukder et al., 2012). When primary pDCs derived from the bone marrow of *Plscr1^-/-^* and *Wt* mice were stimulated with CpG-ODN, HSV or IAV, IFN-α production was significantly reduced in *Plscr1^-/-^* pDCs, while the production of IL-6 and TNF-α was unaffected. This suggests that PLSCR1 and TLR9 interact to regulate the production of type I IFN specifically in pDCs.

TLR7, another PRR located in endosomes, specializes in sensing single-stranded RNA (Heil et al., 2004). Importantly, since IFN-α production by *Plscr1^-/-^* pDCs was impaired in infection not only with HSV (a DNA virus), but also with IAV (an RNA virus), both TLR9 and TLR7 pathways might be affected. Moreover, TLR9 is typically located in the endoplasmic reticulum (ER) at rest and relocates to endosomes following exposure to nucleic acids (Latz et al., 2004). Talukder et al. demonstrated that PLSCR1 was required for the recruitment of TLR9 to the endosome, as TLR9 was absent in endosome in *Plscr1^-/-^* pDCs after CpG-A stimulation (Talukder et al., 2012). Furthermore, the nuclear translocation of IRF7 induced by CpG-A was abolished in the absence of PLSCR1, suggesting that PLSCR1-TLR9 interaction is necessary for all downstream signaling. Given that type I IFNs are central to antiviral immunity by enhancing the responses of T cells, NK cells and B cells (McNab et al., 2015), the interaction between PLSCR1 and TLR9 in pDCs may play a key role in antiviral defense against a broad range of both DNA and RNA viruses.

## 4 Pro-viral activities of PLSCR1

Although numerous studies have documented the antiviral activities of PLSCR1, two reports highlight its role in promoting viral replications (Gong et al., 2011; Cheshenko et al., 2018). First, Gong et al. identified PLSCR1 as a pro-viral attachment factor that facilitates HCV entry into hepatoma cells (Gong et al., 2011). HCV E1 and E2 proteins are important for viral attachment to host cells by binding to several host entry factors, including occludin (OCLN) (Dubuisson, 2007; Ploss et al., 2009). Gong et al. found that PLSCR1 physically interacts with both HCV E1 and E2 proteins and occludin (OCLN) (Gong et al., 2011). Knocking down PLSCR1 in Huh-7.5.1 cells inhibited HCV entry and subsequent replication by preventing initial viral attachment. Therefore, PLSCR1 promotes HCV attachment, entry, and replication by interacting with both viral attachment proteins and host entry factor.

The opposite effect of PLSCR1 was also observed in the case of HSV. Cheshenko et al. found that HSV activates the enzymatic activity of PLSCR1, which in turn translocates Akt, a dock for HSV, to the outer leaflet of plasma membrane, thus promoting viral entry (Cheshenko et al., 2018). HSV binds to host cell membrane nectin-1 through its viral glycoprotein D (gD) (Connolly et al., 2005). In order to fuse with plasma membrane and subsequent entry, viral glycoprotein B (gB) must interact with host Akt, which is normally localized in the cytoplasm and on the inner leaflet of plasma membrane (Cheshenko et al., 2013). In response to a transient Ca^2+^ release following gD-nectin engagement, PLSCR1 undergoes tyrosine phosphorylation as early as 15 minutes post HSV-1 and HSV-2 infections. It then catalyzes the externalization of both PS and Akt in human cervical epithelial cells (CaSki) and keratinocytes (HaCAT) (Cheshenko et al., 2018). Externalized Akt is subsequently phosphorylated by PLSCR1, leading to viral entry and a secondary release of intracellular Ca^2+^ stores. Notably, keratinocytes, which are naturally susceptible to HSV, express much lower levels of PLSCR1 at baseline compared to dendritic cells, which are not the preferred cell type for HSV infection (Karlsson et al., 2021). It can be inferred that the anti- or pro-viral activities of PLSCR1 may not be determined by the specific viral species, as both effects have been observed in HCV and HSV infections. Instead, these conflicting roles are likely due to different underlying mechanisms and infected cell types.

## 5 Conclusions

PLSCR1 is a versatile protein that plays a crucial role in host defense against multiple viruses that are highly pathogenic. From directly interfering with viral replication to modulating key immune pathways, considerable progress has been made in understanding its broad-spectrum antiviral activity. One of the key insights from recent studies is the functional separation between PLSCR1’s enzymatic activity as a phospholipid scramblase and its antiviral functions. While its canonical role involves membrane phospholipid rearrangement, PLSCR1’s ability to regulate gene transcription and interact with viral proteins suggests alternative mechanisms through which it contributes to host defense. This raises important questions regarding the evolutionary adaptation of PLSCR1, which may have evolved from a simple membrane-associated enzyme into a multifunctional immune modulator.

Despite its well-documented antiviral functions, there are instances where PLSCR1 promotes viral replication, as seen in HCV and HSV infections (Gong et al., 2011; Cheshenko et al., 2018). These pro-viral effects highlight the complexity of PLSCR1’s interactions with different viruses and suggest that its role may be context-dependent, shaped by the specific cellular and molecular environments during infection. A deeper understanding of these contrasting roles could help develop strategies to selectively enhance its antiviral functions while mitigating its pro-viral effects.

Another key aspect that warrants further exploration is the relationship between PLSCR1 and apoptosis, particularly in the context of viral infections. While PLSCR1 has been implicated in apoptotic pathways in other diseases, such as glaucoma (Luo et al., 2023), its potential role in virus-induced apoptosis remains largely unaddressed. Many viruses, including IAV (Ampomah and Lim, 2020), HIV (Basanez and Zimmerberg, 2001), and HSV (Wang et al., 2024), exploit apoptosis as a strategy for viral dissemination, promoting the release of progeny virions and facilitating immune evasion. Given that PLSCR1 is involved in PS externalization—a hallmark of apoptosis—it is crucial to examine its role in virus-induced cell death. If PLSCR1 enhances the clearance of virus-infected apoptotic cells, it could serve as a host defense mechanism. However, if PLSCR1 is co-opted by viruses to accelerate apoptosis and immunopathogenesis, it could facilitate viral propagation and viral infection-induced tissue damage. Further research is needed to clarify whether PLSCR1 plays a protective or detrimental role in virus-induced apoptosis and its impact on host-pathogen interactions.

In conclusion, given its ability to restrict virus entry, nuclear localization and transactivation, as well as its role in regulating anti-viral signaling events in both immune and non-immune cells, PLSCR1 emerges as a promising target for anti-viral therapies. Potential therapeutic approaches could include: 1) the development of small-molecule PLSCR1 mimetics designed to interfere with viral entry; and 2) the encapsulation of PLSCR1 in vectors, such as nanoparticles, to achieve targeted delivery for enhanced efficacy. In addition, exploring the synergistic effects between emerging PLSCR1-based therapies and existing antiviral drugs could lead to the development of novel strategies, thereby improving our ability to combat current and future viral infections.
